# Droplet Microfluidics for High-Throughput Analysis of Antibiotic Susceptibility in Bacterial Cells and Populations

**DOI:** 10.1021/acs.accounts.1c00729

**Published:** 2022-02-04

**Authors:** Witold Postek, Piotr Garstecki

**Affiliations:** Institute of Physical Chemistry, Polish Academy of Sciences, ul. Kasprzaka 44/52, 01-224 Warszawa, Poland

## Abstract

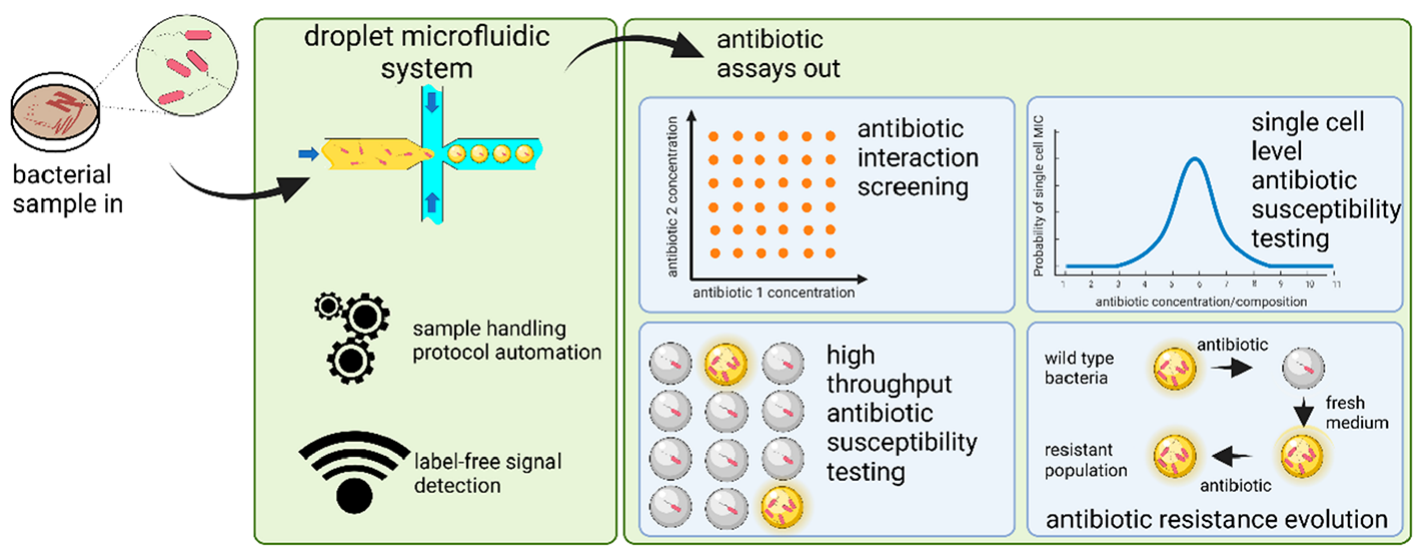

Antibiotic-resistant bacteria are an increasing concern both in everyday life and specialized environments such as healthcare. As the rate of antibiotic-resistant infections rises, so do complications to health and the risk of disability and death. Urgent action is required regarding the discovery of new antibiotics and rapid diagnosis of the resistance profile of an infectious pathogen as well as a better understanding of population and single-cell distribution of the resistance level. High-throughput screening is the major affordance of droplet microfluidics. Droplet screens can be exploited both to look for combinations of drugs that could stop an infection of multidrug-resistant bacteria and to search for the source of resistance via directed-evolution experiments or the analysis of various responses to a drug by genetically identical bacteria. In droplet techniques that have been used in this way for over a decade, aqueous droplets containing antibiotics and bacteria are manipulated both within and outside of the microfluidic devices. The diagnostics problem was approached by producing a series of microfluidic systems with integrated dilution modules for automated preparation of antibiotic concentration gradients, achieving the speed that allowed for high-throughput combinatorial assays. We developed a method for automated emulsification of a series of samples that facilitated measuring the resistance levels of thousands of individual cells encapsulated in droplets and quantifying the inoculum effect, the dependence of resistance level on bacterial cell count. Screening of single cells encapsulated in droplets with varying antibiotic contents has revealed a distribution of resistance levels within populations of clonally identical cells. To be able to screen bacteria from clinical samples, a study of fluorescent dyes in droplets determined that a derivative of a popular viability marker is more suitable for droplet assays. We have developed a detection system that analyzes the growth or death state of bacteria with antibiotics for thousands of droplets per second by measuring the scattering of light hitting the droplets without labeling the cells or droplets. The droplet-based microchemostats enabled long-term evolution of resistance experiments, which will be integrated with high-throughput single-cell assays to better understand the mechanism of resistance acquisition and loss. These techniques underlie automated combinatorial screens of antibiotic resistance in single cells from clinical samples. We hope that this Account will inspire new droplet-based research on the antibiotic susceptibility of bacteria.

## Key References

- Churski, K.; Kaminski, T. S.; Jakiela, S.; Kamysz, W.; Baranska-Rybak, W.; Weibel, D. B.; Garstecki, P. Rapid screening of antibiotic toxicity in an automated microdroplet system. Lab Chip2012, 12, 1629–1637.2242217010.1039/c2lc21284f(1)*Droplet-based study of interactions between antibiotics run in an automated fashion. We generated a sequence of droplets with different ratios of two antibiotics with bacteria added after which we monitored the growth of encapsulated bacteria.*
- Postek, W.; Gargulinski, P.; Scheler, O.; Kaminski, T. S.; Garstecki, P. Microfluidic screening of antibiotic susceptibility at a single-cell level shows the inoculum effect of cefotaxime on *E. coli*. Lab Chip2018, 18, 3668–3677.3037560910.1039/c8lc00916c(2)*Quantification of the inoculum effect and identification of the scMIC level in an automated fashion with emulsions separated in flow. We observed single cells within a clonal population with varying levels of antibiotic resistance.*
- Scheler, O.; Makuch, K.; Debski, P. R.; Horka, M.; Ruszczak, A.; Pacocha, N.; Sozański, K.; Smolander, O.-P.; Postek, W.; Garstecki, P. Droplet-based digital antibiotic susceptibility screen reveals single-cell clonal heteroresistance in an isogenic bacterial population. Sci. Rep.2020, 10, 3282.3209449910.1038/s41598-020-60381-zPMC7039976(3)*Quantification of the scMIC distribution within a clonal population. We calculated the critical number of antibiotic molecules per bacterial cell necessary to stop colony growth in droplets.*
- Jakiela, S.; Kaminski, T. S.; Cybulski, O.; Weibel, D. B.; Garstecki, P. Bacterial growth and adaptation in microdroplet chemostats. Angew. Chemie - Int. Ed.2013, 52, 8908–891110.1002/anie.201301524PMC387916023832572.^4^*Long-term cultivation of bacteria in microliter droplets coupled with directed evolution of antibiotic resistance. We periodically exchanged spent medium from droplet microchemostats with fresh medium with increasingly concentrated antibiotic.*

## 1 Introduction

Antibiotic resistance is a major threat to public health worldwide.^5^ Multidrug-resistant superbugs jeopardize surgeries or immunosuppressive therapies, threatening a large fraction of current medicine and the human lifespan across countries and economies. The COVID-19 pandemic forced many countries into prolonged lockdowns, causing significant economic downturn and killing almost 4.5 million people worldwide at the time of submission of this Account after less than 2 years of the pandemic. Meanwhile, antibiotic-resistant diseases are estimated to cause ca. 700 000 deaths per year; the WHO envisions 10 million deaths per year attributable to antimicrobial-resistant infections by the year 2050.

Antibiotic-resistant strains of bacteria were discovered soon after the introduction of antibiotics. Alexander Fleming mentioned this in his Nobel lecture in 1945: *It is not difficult to make microbes resistant to penicillin in the laboratory by exposing them to concentrations not sufficient to kill them, and the same thing has occasionally happened in the body*. Using wrong antibiotics or even right antibiotics at the wrong dose produces resistant bacteria via natural selection. New methods of rapid antibiotic susceptibility testing (AST) are urgently needed. Droplet-based microfluidics may be an alternative (or at least a supplement) to common low-throughput antibiotic resistance tests and could open new paths for research into antibiotic resistance at the population and single-cell levels.

Droplet microfluidics, where droplets of one liquid (e.g., water) are submerged in another liquid (e.g., oil), enables large numbers of tests, because millions of droplets can be easily generated, and each droplet can contain a slightly different reagent composition^6^ and/or contain an individual object of interest such as a single cell or a single piece of target nucleic acid for amplification (PCR, LAMP, etc.). Droplets can be controllably merged, split, or sorted on the basis of droplet fluorescence^7^ or absorbance.^8^ Run-of-the-mill droplet generators are straightforward in design and readily available from commercial vendors; however, their proper use requires additional equipment, such as pressure generators for pushing liquids around channels (typically achieved with syringe pumps) or custom-made optical setups.

In this Account, we guide the reader through advances in the field of droplet-based AST. We first describe how the AST routines can be automated within microfluidic devices in a variety of ways. We then proceed to how the AST can be brought to the level of single cells by means of droplets. In this section, we highlight how the analysis of single cells can help avoid an error-generating inoculum effect and what additional information can be extracted from an AST assay when the assay is single-cell oriented in comparison to a classical population-level assay. We also describe the hurdles of bacterial growth detection in droplets at high throughput and what can be done to avoid or overcome these issues. Then, we describe how long-term studies of bacterial evolution can be run within droplets with automated protocols. We then arrive at the conclusions where we mention problems to be solved within the field of droplet AST, and we draft possible future research directions.

## 2 Automation Protocols for Antibiotic Studies

Droplet-based assays allow for high-level automation of liquid handling protocols. Here, we describe two different approaches to automating droplet-based antibiotic interaction screening: one based on flowing droplets and another based on printing droplets containing different antibiotics onto a microchamber array.

### 2.1 Droplet-Based Antibiotic Interaction Screening

**Scheme 1:**
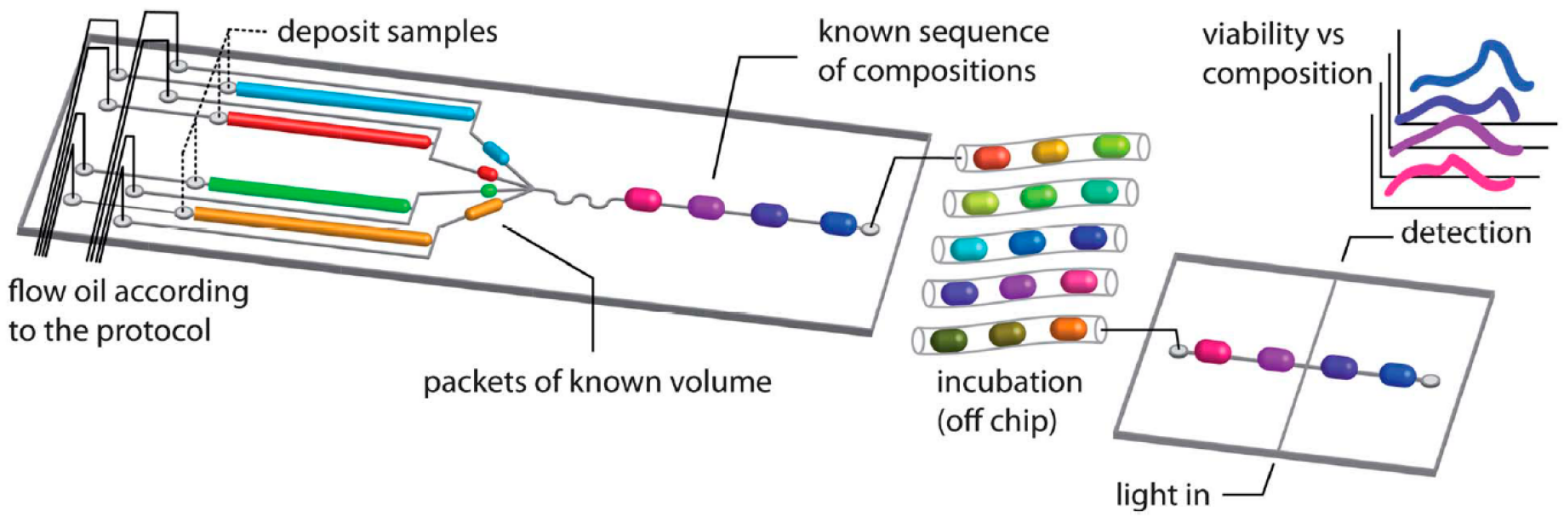
Generation of Sequences of Droplets with Controllable Contents Samples are precisely portioned on a microfluidic chip and mixed to form a droplet of a desired composition. Droplets can be incubated to stimulate the growth of encapsulated cells and later optically screened for growth to link a sample’s composition with a cell’s behavior. Reproduced with permission from ref (1). Copyright 2012 The Royal Society of Chemistry.

To quantify antibiotic resistance, the minimum inhibitory concentration (MIC) is measured by preparing a serial dilution of a tested antibiotic and inoculating it with a known and constant density of bacteria.^9^ After hours of incubation at 37 °C, samples are analyzed and the lowest concentration of the drug in which no bacterial growth is recorded is established as the MIC of a pair of a given antibiotic and a given bacterial strain. Screening bacteria for resistance to two antibiotics by establishing the MIC in 10 different concentrations of each antibiotic requires the preparation of 100 dilutions, a time-consuming task. Utilizing on-demand generation of nanoliter-sized droplets,^10^ we designed a system that automatically prepared combinations of two drugs at various concentrations^1^ (Scheme 1).

**Figure 1:**
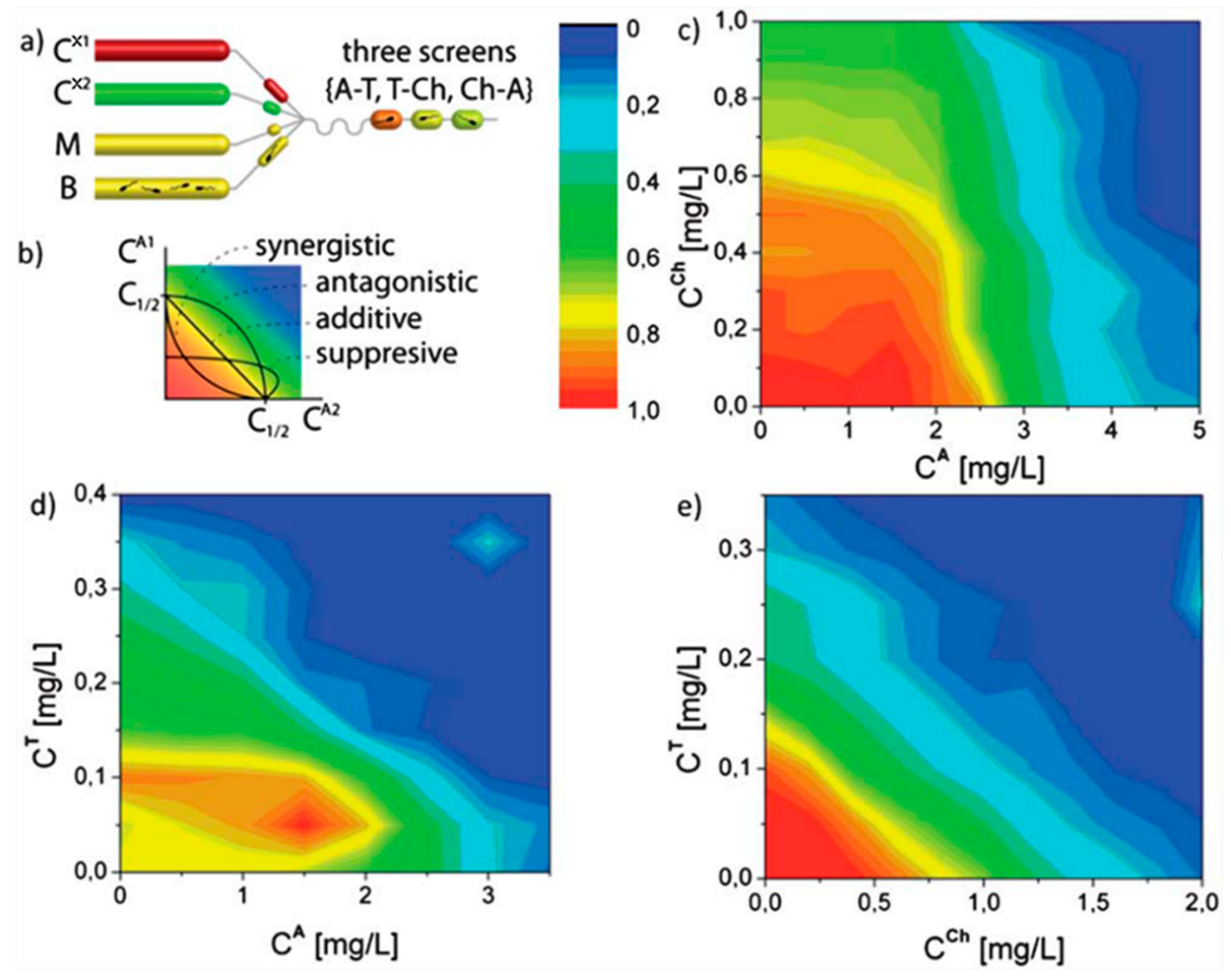
Interaction of two antibiotics as measured in an automated fashion in a microfluidic device. (a) Droplets were generated to run antibiotic resistance screens with antibiotics tetracycline (T), chloramphenicol (Ch), and ampicillin (A). The drugs were used at concentrations *C*^X1^ for one antibiotic and *C*^X2^ for another antibiotic. The antibiotic droplets were merged with droplets of a growth medium (M) and bacterial suspension (B). (b) The antibiotic interactions are described on the basis of the definition of Loewe additivity^12^ and identified by the shape of the isobole (line of constant inhibition) in the plots of the two-dimensional matrices of cell viability (c–e). The data points from these plots of cell viability (c–e) imply droplets with different ratios of two given antibiotics. Warm colors mean high fluorescence intensity of resorufin in the droplets, implying the growth of bacteria in the droplets, while cool colors mean no growth. Adapted with permission from ref (1). Copyright 2012 The Royal Society of Chemistry.

After incubating the generated droplets, interactions were established between drugs (synergistic, antagonistic, or additive).^11,12^ Samples were diluted by mixing an antibiotic sample with pure medium so that after each mixing event the final volume of the droplet would be the same. This assay allowed us to identify interactions between antibiotics at a high resolution of antibiotic concentrations (Figure 1). The concentration was controlled by changing the ratios of volumes of sample and medium (Scheme 1, Figure 1a). We quickly determined that the system was not suitable for screening clinical samples: the dilution method generated concentrations only in a small range, which is not useful when the MIC of a bacterium is not known, as with clinical samples. A method of diluting antibiotics with a broad dynamic range was therefore required. We have developed such a method with flowing droplets;^13^ however, this particular solution was highly complicated and thus not easily translatable to a clinical setting. We have therefore explored off-chip dilution of samples.

### 2.2 Printing Droplets into Microfluidic Devices for Antibiotic Interaction Screening at a Broad Range of Antibiotic Concentrations

**Figure 2:**
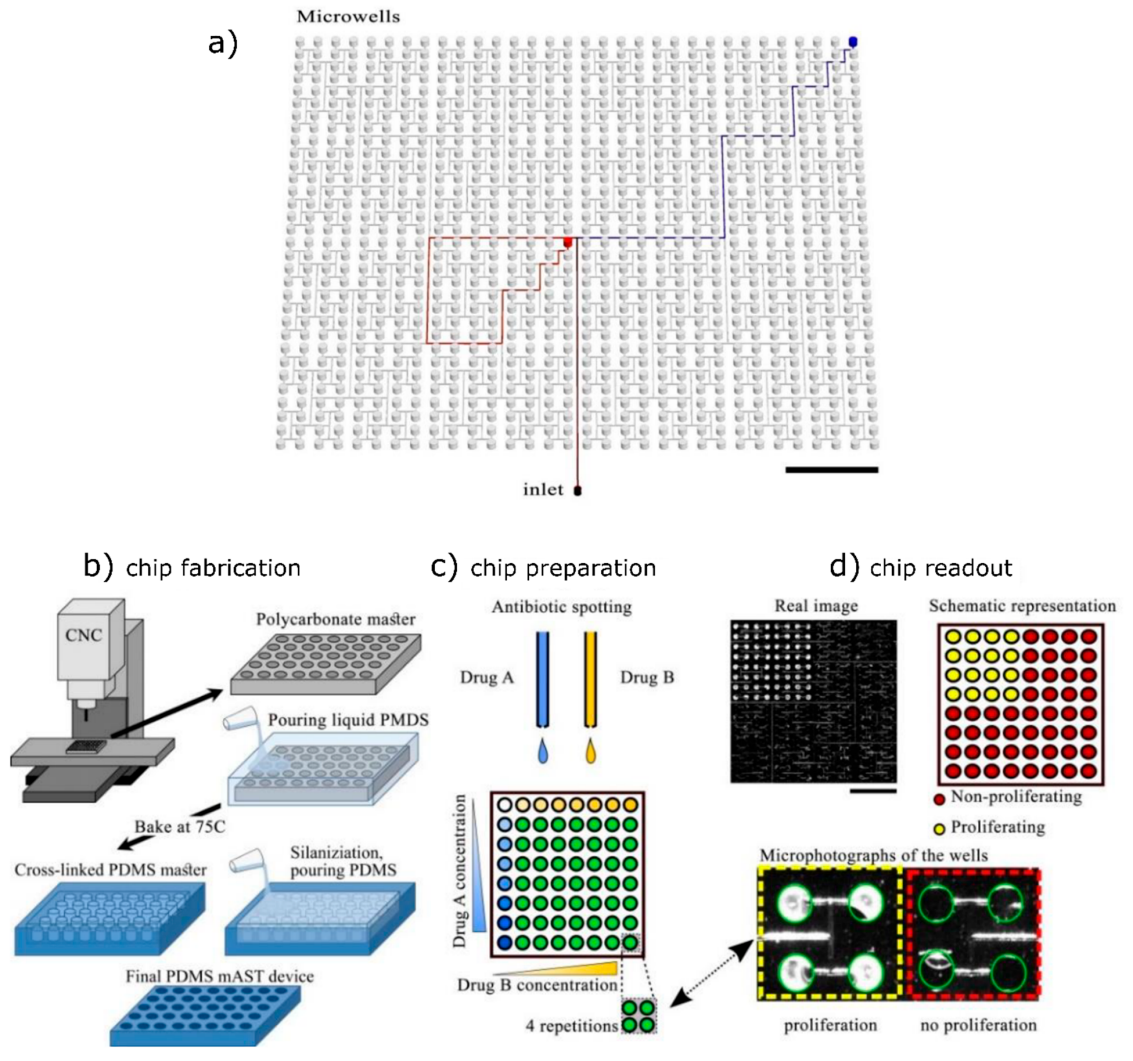
(a) Microwells for antibiotic interaction studies are connected to the inlet port by equidistant channels, providing the same hudraulic resistance on the way to each well and thus ensuring equal filling of the wells with liquids. The wells are prefilled with antibiotics via droplet printing, and the solvent is left to dry. Then, after the microwell array is closed, the bacteria and medium are added by the inlet port to the channels and separated by fluorinated oil. (b) The microwell plate is made of PDMS based on a CNC-milled master. (c) Microwells are filled with antibiotics in different concentrations by a noncontact printer. (d) Top left: real image of the microwell array after growth of the bacteria; top right: binary representation of the wells with or without bacterial proliferation; bottom: close-up of the wells with proliferating or nonproliferating bacteria. The final device contained 1024 wells. Adapted with permission from ref (14) under the Creative Commons BY license. Copyright 2020.

Off-chip dilution for sample preparation allows for one to use large and precise equipment before placing the diluted samples onto a small and disposable chip. In our solution, various numbers of antibiotic molecules and different antibiotics were printed into >1000 nanoliter-sized chambers to screen for resistance to cocktails of antibiotics (Figure 2).^14^ When a bacterial solution is pipetted into the chip with printed antibiotics, the liquid is sucked into the chambers as the chip tries to refill its polymer structure with gas as the chip had been degassed before the experiment. As the channels branch fractally from the inlet, there is no pressure difference between chambers and the filling is accurate. We can monitor the reaction outcome by absorbance readout. The current focus is on using disposable pumps that make the degassing of printed chips unnecessary. Printed devices offer large experimental scale and multiplexing of reaction conditions versus other chamber-based systems in which each set of chambers requires manual dilution of a drug.^15,16^

## 3 Droplets for Single-Cell Antibiotic Studies

Microfluidics is perfect for single cell studies, as individual organisms can either be placed in structures that would fit only a single cell^17^ or cells can be placed in individual droplets.^18^ Fitting cells in a single-phase microfluidic device is similar to organizing objects under a microscope with the addition of excellent fluid control: cells are immobilized, can be easily tracked, and can be fed with nutrients or antibiotics. Tracking individual cells allows for a rapid phenotypic MIC assay, as the cells can be monitored to determine whether they divide in the presence of an antibiotic.^19^ Scaling up such an assay with a mother machine enabled an even faster, statistics-based MIC assay.^20^ A mother machine is a widespread microfluidic device that consists of a main deep channel and shallow dead-end channels perpendicular to the main channel. Each of the shallow channels can trap a single bacterium that does not escape as the shallow channels are dead-end. After each division of the trapped cell, the daughter cells appear closer to the main channel, but the original mother cell stays always at the end of the shallow channel. A mother machine used for AST allows one to monitor multiple individual cells and their growth dynamics under antibiotic stress.^20^ The addition of microvalves to a mother machine’s shallow channels allows for one to distinguish between two species of bacteria used in an assay in addition to assessing their MIC.^21^ Although impressive, these single-phase solutions are still orders of magnitude below the scale of droplet-based cell assays (e.g., 10^7^ droplets were used per experiment to look for rare mutants highly resistant to antibiotics,^22^ or tens of thousands of droplets with various antibiotics at different concentrations were placed in wells to look for MICs of cocktails of antibiotics^23^).

### 3.1 Droplet Libraries for Single Cell Studies

The historically first system for AST in droplets used an off-chip dilution protocol: a series of drug concentrations was prepared with a pipet and then portions of these prepared solutions were aspirated to a piece of tubing by a syringe pump, separated by an air spacer.^24^ The drug-containing droplets were merged on-chip with solutions of bacteria and a bacterial viability indicator. After merging, the samples were emulsified into 50 droplets each and incubated for growth of the bacteria that would later be detected optically. The separation of the emulsions with air allowed for the identification of reaction conditions in a given set of 50 droplets. Each droplet contained at most a single bacterial cell at the beginning of the experiment. While this work was promising in terms of studies of the antibiotic resistance of single cells, 50 droplets per experiment are not sufficient to achieve statistical power; to encapsulate single cells in the droplets, one has to dilute the sample before emulsification. According to Poisson statistics, to distribute cells among droplets so that only a very small portion of droplets with bacteria contain more than one cell, the majority of generated droplets has to be empty.^25^ If there are 10 cells in a sample that is divided into 100 droplets, there is only a small chance that any droplet will end up with 2 or more cells. Thus, in the assay described above,^24^ 50 droplets per experiment yielded only 5 droplets with single cells per experiment.

**Scheme 2:**
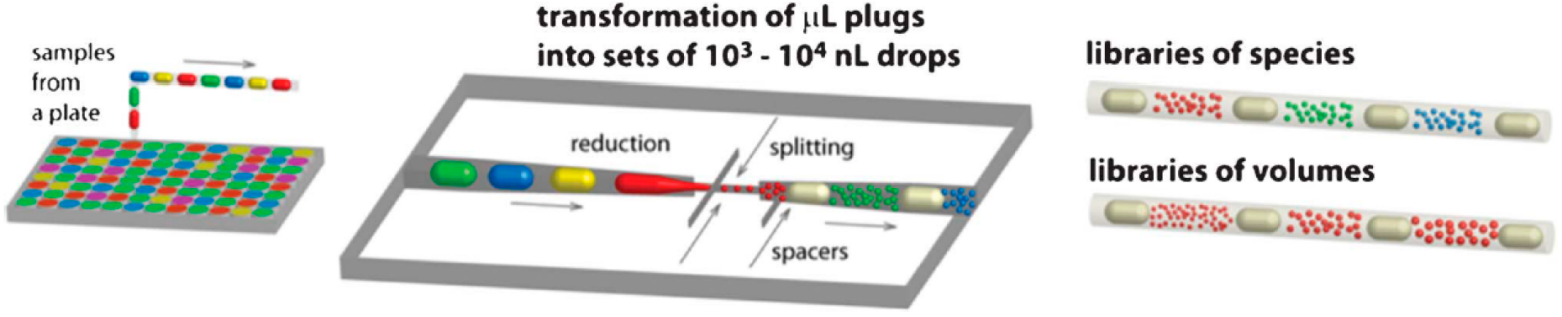
Generation of Droplet Libraries for Multiparameter Single-Cell Screening Samples can be prepared on a well plate manually or with a fluid-handling robot and then automatically transferred to the microfluidic device. Each sample is partitioned into hundreds or thousands of smaller droplets and separated from each other with a spacer. Each library contains different reagent compositions, and each droplet within a library can be designed to contain no more than a single cell. Reproduced with permission from ref (26). Copyright 2012 The Royal Society of Chemistry.

Serial dilutions that we perform on-chip are prepared in large (submicroliter) droplets, but it is also possible to prepare a series of dilutions of antibiotics off-chip and then emulsify the diluted samples (Scheme 2). The latter method would achieve a multiplexed assay with a broad dynamic range and would produce either (i) thousands of experimental replicates (as each droplet in a given emulsion would be a replicate) or (ii) individually encapsulated cells for study at high throughput.^26^

### 3.2 High-Throughput Droplet Libraries

To improve on the historic system,^24^ we experimented with generating “libraries” of droplets. A series of large sample droplets (“mother droplets”) was prepared and then emulsified; each emulsion with a different set of reagents constituted a separate library.^26,27^ We developed systems for both active and passive formation of multiple emulsions.^2,26^ Active emulsification with a module called *flow-focusing* quickly generates hundreds or thousands of droplets but requires a large proportion of oil (the continuous phase) to water (dispersed) to generate an emulsion.^26^ Passive emulsification generates droplets more slowly but without the excess of a continuous phase in a mechanism called microfluidic step emulsification (MSE).^28^ We employed MSE to screen for antibiotic resistance of single cells^2^ by generating multiple emulsions in series, each with a different antibiotic concentration. The emulsions had to be identifiable in order to link any given droplet with a particular antibiotic concentration. Although color-coding of droplets (marking droplets with unique sets of concentrations of distinct fluorescent dyes) has been successfully used in a microfluidic AST,^23^ a label-free system like ours would be beneficial to reduce the cost of the method, as each color for coding requires separate excitation light.

**Figure 3:**
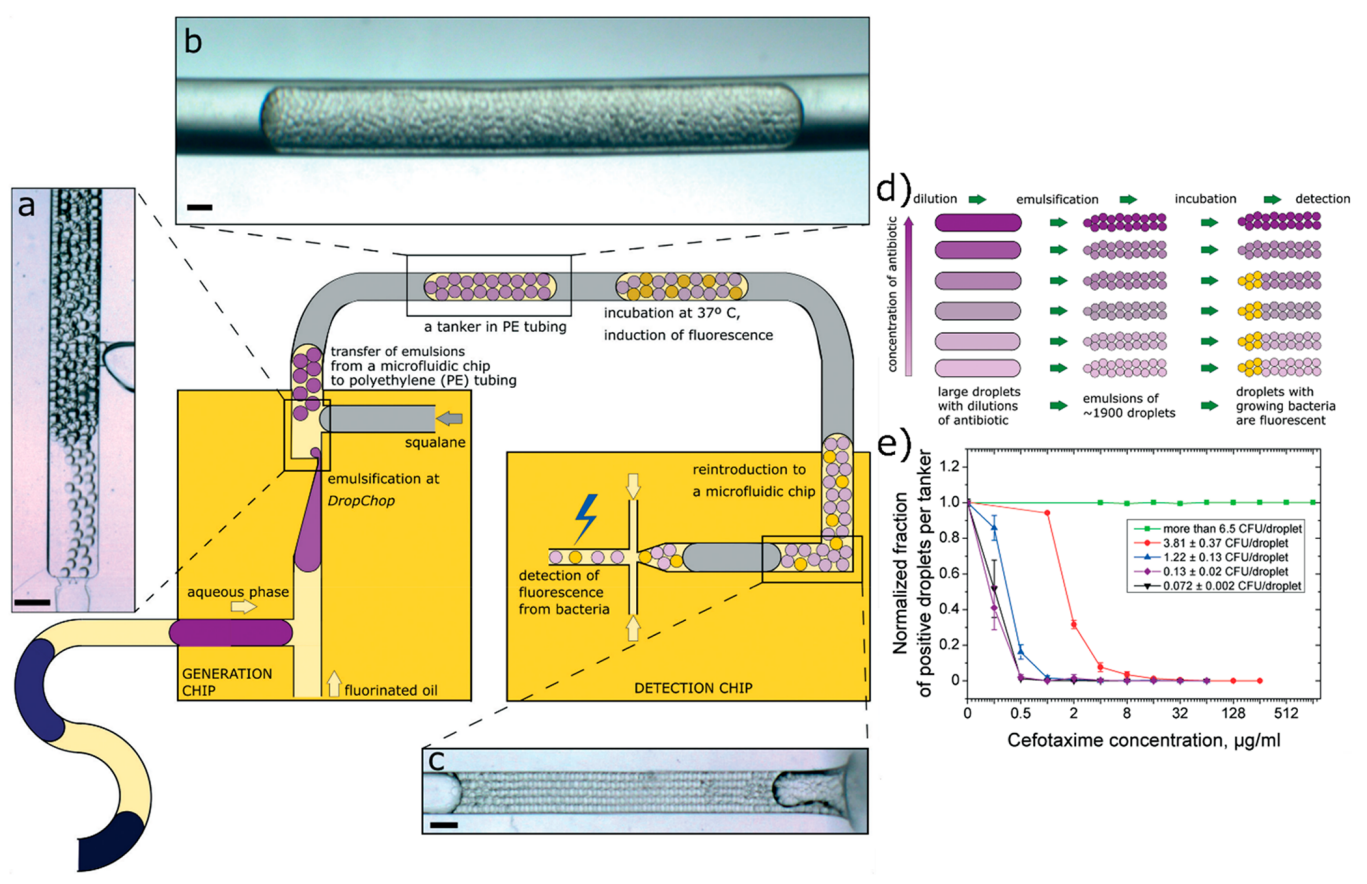
Single-cell minimum inhibitory concentration (scMIC) tanker assay. (a–c) Generation of tankers, emulsions with different reagent compositions separated in flow with an oil spacer. (d) concept of scMIC experiments in emulsions. (e) scMIC measurements acquired with the tanker system. The inoculum effect is immediately visible: with more bacterial cells per droplet, the antibiotic concentration needed for growth inhibition is higher. scMIC is the smallest measured MIC, as in another study done in bulk volumes.^30^ Adapted with permission from ref (2). Copyright 2018 The Royal Society of Chemistry.

We physically separated emulsions of different drug concentrations from each other, which would be inefficient if every generated emulsion was kept in a separate well on a plate. To keep the emulsions in flow, after generating each emulsion of aqueous droplets submerged in fluorinated oil, we injected a portion of hydrocarbon oil into our system to separate the emulsions (Figure 3). While this new solution was similar to the classic solution,^24^ using oil instead of air to separate emulsions with proper oil and chip construction materials ensured that emulsions did not wet the incubation channel walls, preventing the droplets from traveling between emulsions during experiments and causing cross-contamination. As MSE was used to generate droplets, each emulsion was densely packed. Emulsions separated from each other with oil are called *tankers*. Tankers were incubated to let bacteria grow and give off a fluorescent signal that we could detect. The tanker-based system allows one to screen for resistance with multiplexing of the reaction conditions because a robotic positioning system is used to generate mother droplets with various antibiotic concentrations. Similar multiplexing can be achieved with modified plates for running reactions,^19^ printing antibiotics on chip,^14^ or using complex integrated systems.^29^ If the throughput of the tanker system is increased, the system should allow one to screen dozens of reaction conditions per experiment, yielding screening of antibiotic cocktails at the level of single cells, which has not yet been achieved in an automated way.

### 3.3 Inoculum Effect in AST Can Be Alleviated by Isolating Single Cells in Droplets

In the system presented in Figure 3, we used a range of initial concentrations of bacteria in the sample such that isolated single *Escherichia coli* cells and populations with >5 cells were tested against the antibiotic cefotaxime. It was assumed that, for an emulsion with no antibiotic, every droplet that contained cells provided for the growth of bacteria and gave off a fluorescent signal from constitutively expressed yellow fluorescent protein (YFP). The fraction of droplets that yielded a signal (positive fraction) was divided by the total number of droplets for each emulsion and then was normalized against the no-antibiotic control, thus generating data points for viability curves (the percentage of droplets that provided growth in the increasing antibiotic concentration). The MIC was higher for the higher initial concentrations of bacteria in the sample (Figure 3e), a phenomenon called the inoculum effect that was recently shown to be important in the evolution of resistance^30^ and to be the cause of considerable errors of measurements of MIC in the clinic.^31^

### 3.4 Droplet-Based Screening of Isogenic Cells Shows Antibiotic Resistance Level Variation within Populations

**Figure 4:**
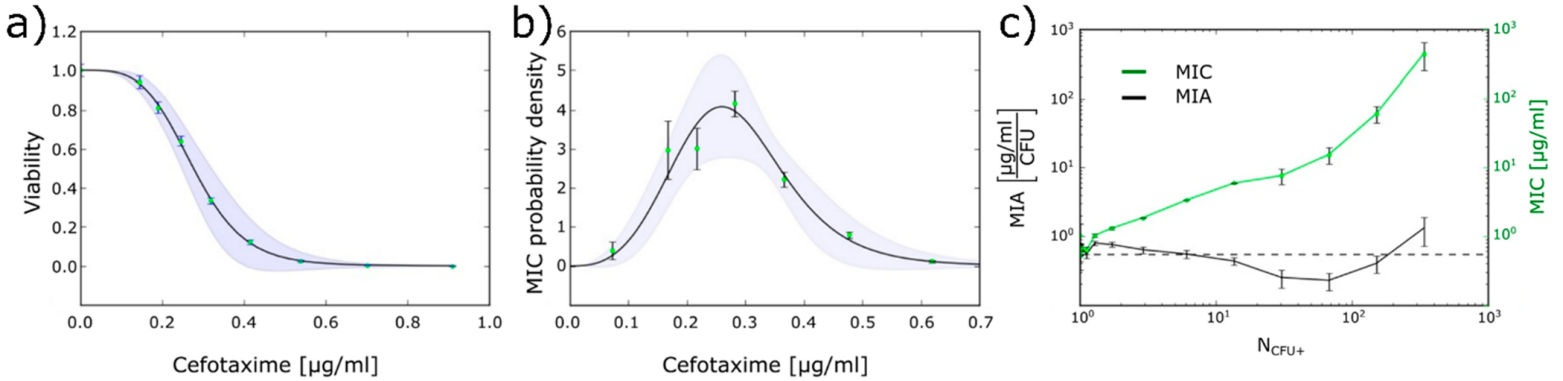
Single cell-level resistance screens in droplets. (a) Viability curve of *E. coli* acquired from droplet-based single cell experiments. (b) Probability distribution of scMIC based on the curve from (a). The shaded area shows errors obtained from the error propagation formula applied to the negative derivative of fit from (a). (c) Minimal inhibitory concentration (MIC) and minimal inhibitory amount (MIA) measured for different inoculum densities (*N*_CFU+_). MIA is defined as the number of antibiotic molecules per bacterium normalized to droplet volume. Adapted from ref (3) under a Creative Commons Attribution 4.0 International License. Copyright 2020.

As seen in Figure 3e, at a low antibiotic concentration for droplets only containing a single cell, roughly half the droplets supported the growth of bacteria and half the droplets did not. In a subsequent study,^3^ we focused on the range of concentrations of cefotaxime that produced heterogeneous results in the previous assay.^2^ We produced viability curves for single cells by fitting a Gompertz function to positive droplet fractions (Figure 4a), and by taking a derivative of this function, we determined the distribution of the probability that a given cell has a given scMIC (Figure 4b). In other words, a distribution of antibiotic resistance in an isogenic population of cells was obtained. It is unclear whether this distribution arises from stochasticity in gene expression, in which a gene encoding resistance (in this case, TEM-20 β-lactamase) randomly has higher or lower expression in each cell, or if it is the distribution of the copy number of the plasmid harboring the resistance gene that influences the resistance-level distribution within the population. Experiments are currently performed on bacteria with resistance enzymes encoded on the chromosome instead of on a plasmid to establish the influence of plasmid copy number. In the same study,^3^ we discovered that, although there was a strong inoculum effect, when the number of antibiotic molecules in the sample was divided by the number of bacterial cells in the initial sample, the result was a constant (Figure 4c); the emerging hypothesis is that the number of antibiotic molecules per bacterial cell in the sample determines the MIC of the whole sample, similarly shown for antimicrobial peptides.^32^

### 3.5 Optical Detection of Bacterial Growth within Droplets

The aim of microfluidic AST is to be used in a clinical setting, so ways to identify bacterial growth without labeling the bacteria must be determined. Individual cells can be tracked,^19−21,33^ but while this method can produce results as fast as 30 min,^20^ the scalability of such assays is limited. Optical density-based droplet MIC assays are not rapid because the droplet must become optically dense enough (due to bacterial growth) for detectors to notice at high throughput.^23,34^ Other solutions use proxies of bacterial growth to asses bacterial viability (e.g., measurements of bacterial metabolism,^35^ sample impedance,^36^ or increases in the amount of bacterial DNA in the sample as bacteria grow^37^). The DNA-based MIC assay is pheno-molecular, as it combines gold-standard phenotypic assays with a molecular approach. Without the phenotypic aspect, the genotypic MIC screen is rather prone to errors, as the simple lack of resistance genes is not a valid predictor of antibiotic susceptibility.^38^ Pheno-molecular droplet assays were used to assess the antibiotic susceptibility of clinically acquired bacteria within 30 min of a patient’s sample collection.^37^

#### 3.5.1 Fluorescent Dyes May Leak between Droplets during Incubation

**Figure 5:**
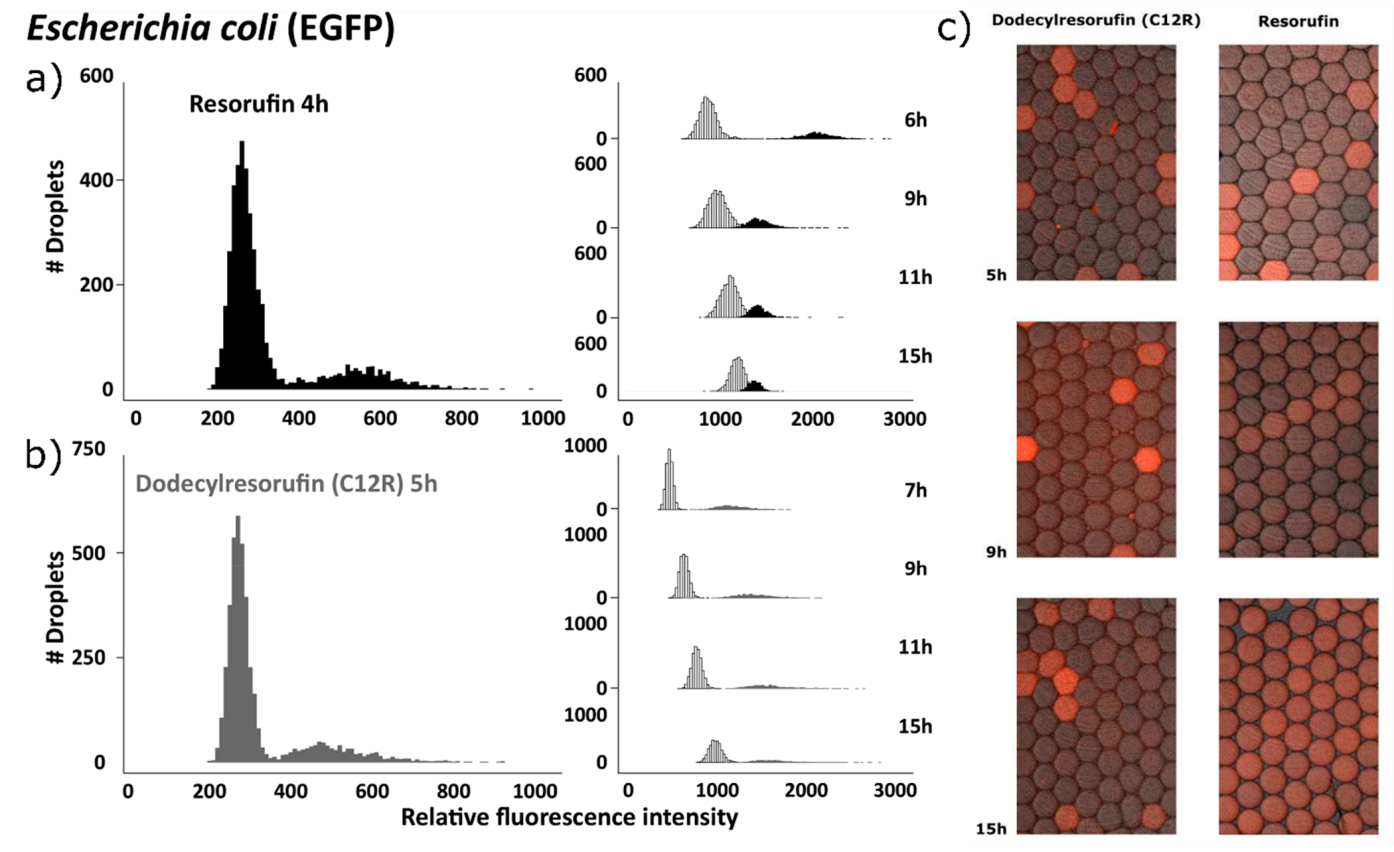
Fluorescent dye leakage from droplets. (a, b) Each of the two fluorescence intensity histograms shows two droplet populations: the more numerous population of negative droplets (not containing living bacteria and thus not fluorescent) and the smaller positive droplet population (containing living bacteria and thus fluorescent). (a) The time-resolved change in fluorescence intensity of a droplet population when resorufin was employed to detect live bacteria, while in (b), dodecylresorufin was the viability marker. Resorufin fluorescence intensity populations merge after 9 h and positive/negative populations become indistinguishable, while dodecylresorufin provides distinct fluorescence intensity of droplet populations even after 15 h of incubation. The fluorescence intensity of negative populations increases over time as a result of the leakage of fluorescent dye from the positive droplet populations, which decrease in fluorescence intensity at the same time. (c) Positive and negative resorufin droplets are indistinguishable after several hours, while dodecylresorufin droplets form clearly distinct populations after 15 h. Adapted from ref (44). Copyright 2016 American Chemical Society.

Even though we have experience in assessing bacterial growth in droplets by measuring droplet oxygen concentration as a proxy of bacterial metabolism,^39^ we turned to optical detection of bacterial growth. Optical detection methods are compatible with high-throughput screening of droplets (thousands of droplets per second),^40,41^ and droplet-based assays can be relatively easily multiplexed.^2,23,29^ Resazurin is a handy replacement for genetically encoded fluorescent proteins and is reduced by metabolically active bacteria to become resorufin. Resorufin becomes fluorescent when exposed to green light, as opposed to an unreduced resazurin. There are two issues with resazurin/resorufin in droplet-based bacterial assays: resorufin requires oxygen-metabolizing bacteria to glow, and resorufin tends to leak from aqueous droplets to surrounding oil (Figure 5). The first issue is not insurmountable, as a major portion of infections in hospitals of developed countries are caused by facultatively anaerobic *E. coli* in urinary tracts. The latter issue is much more devastating: the dye leaks from droplets to oil and neighboring droplets, and it is impossible to determine which droplet contains metabolically active bacteria. Transfer of matter between emulsion droplets is a known problem, especially for the most popular fluorinated surfactant.^42^ Researchers have recently developed new surfactants^43^ or used amphiphilic nanoparticles to form leak-free pickering emulsions.^22^ We replaced resorufin with a different dye, dodecylresorufin (C12R), which is better suited for droplet assays than resorufin; C12R-supplied droplets produce a fluorescent signal above background after hours of incubation, as opposed to leaking resorufin.^44^

#### 3.5.2 Scattering-Based Droplet Screening Allows for Label-Free Detection of Bacterial Growth within Droplets

**Figure 6:**
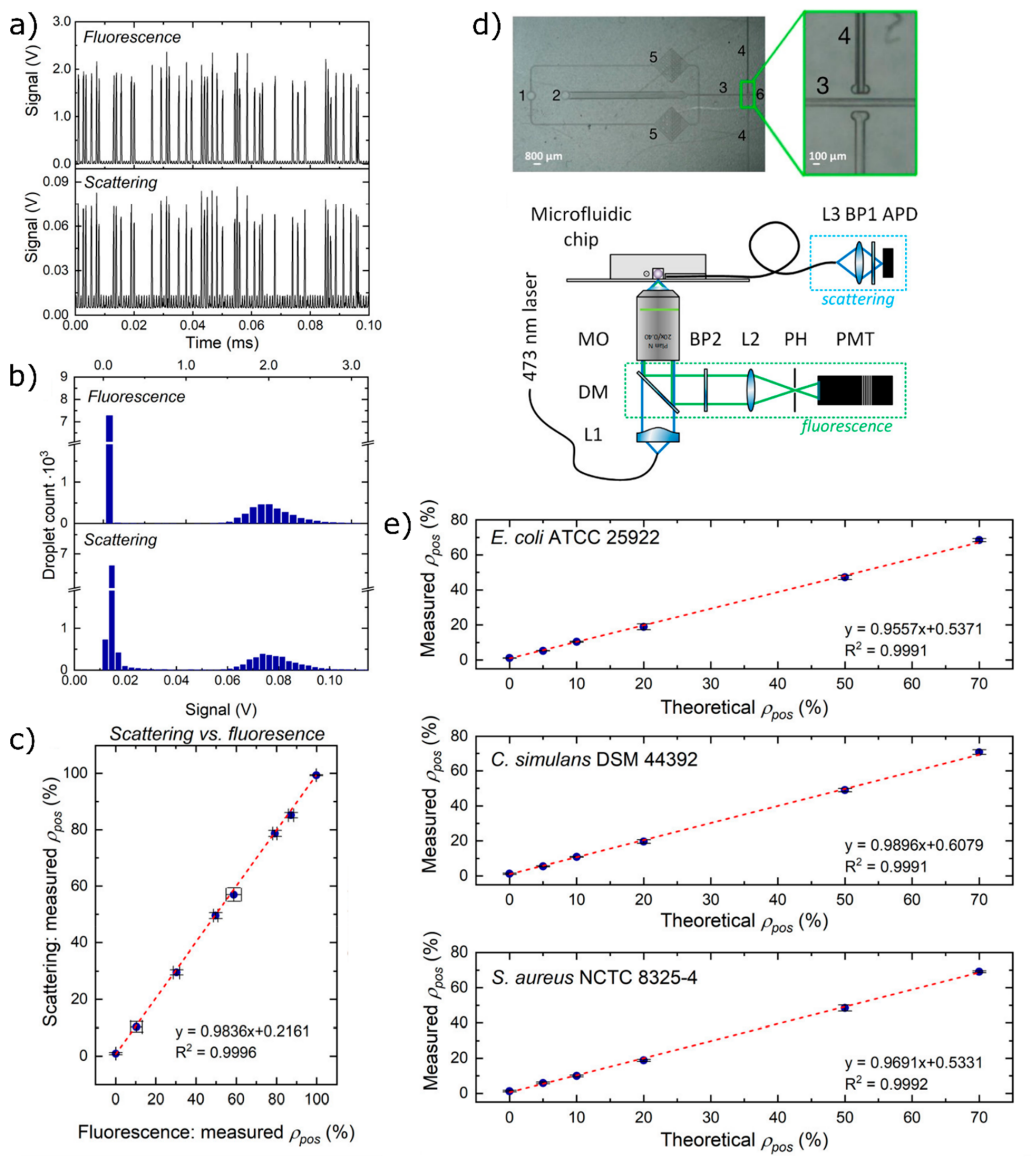
Fluorescence- and scattering-based detection in droplets at high throughputs. (a) Each voltage peak represents a droplet flowing through a detector. (b) High-voltage peaks are positive droplets that form populations clearly distinct from negative droplets for both fluorescence and scattering measurements. (c) Percentages of positive droplets measured by two methods for a strain of bacteria with a fluorescent protein encoded. (d) Top: micrograph showing the microfluidic chip with the flow-focusing junction for droplet screening: 1, inlet for continuous phase; 2, inlet for droplets; 3, detection channel; 4, guiding channels for optical fiber; 5, filters; 6, outlet. Bottom: schematic of the optical setup: L1, lens no. 1; L2, lens no. 2; L3, lens no. 3; DM, dichroic mirror; PH, pinhole; PMT, photomultiplier; MO, microscope objective; BP1, bandpass filter no. 1; BP2, bandpass filter no. 2; APD, avalanche photodiode. (e) Correlation between measured percentages of positive droplets from populations of droplets containing different exemplary nonfluorescent bacterial strains and a theoretical percentage of positive signals from a given dilution of bacteria. Adapted from ref (41). Copyright 2021 American Chemical Society.

We explored light scattering-based detection of growth in droplets as a label-free method (Figure 6).^41^ The light scattered by the bacteria in the droplets changes the direction of propagation and can be gathered with an optical fiber perpendicular to the laser beam. The intensity of the scattered light increases with the number of bacteria so we can distinguish between droplets in which bacteria did not grow and droplets in which bacterial growth was present (Figure 6a,b). To validate the scattering detection system, we paired it with a well-established fluorescence detection system to check if we can gather the same information on bacterial growth from two methods; we used a bacterial species engineered to produce fluorescent proteins for this goal (Figure 6c). As the validation was successful, we proceeded to use only scattering and no fluorescence to detect the growth of 11 different bacterial species that were unmodified genetically and thus not fluorescent (Figure 6e). Our system screens droplets at 1.2 kHz, which is a vast improvement over previously presented 243^34^ or 40 Hz^45^ and is capable of detecting the growth of bacteria from different species. Such a high detection frequency is necessary when looking, for example, for mutants with increased antibiotic resistance, a feature that appears in one per millions of cells.^22^

## 4 Droplet-Based Evolution Screening

**Figure 7:**
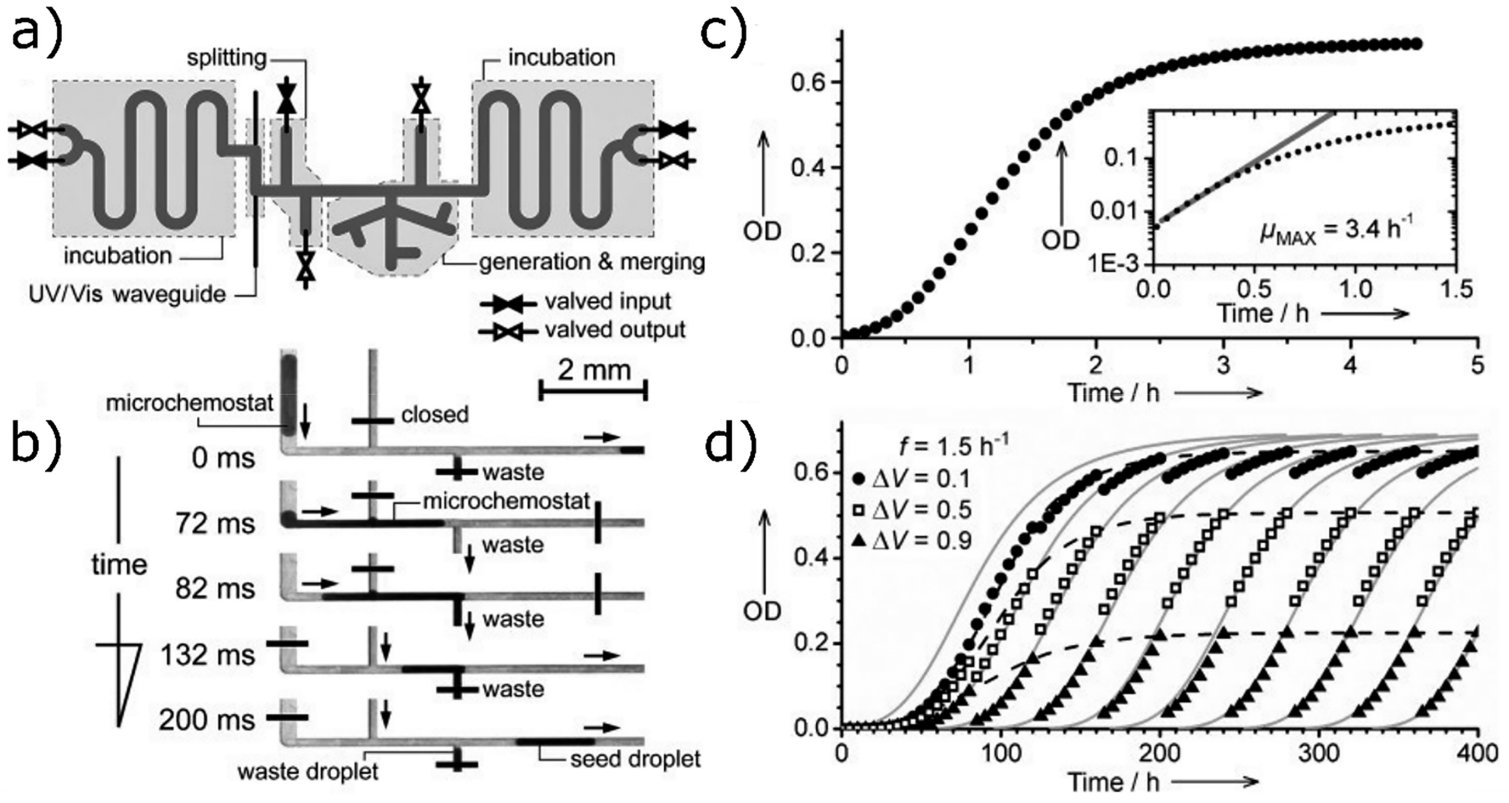
(a) Scheme of a microdroplet chemostat generator, containing multiple valves and channels grouped into sections of different functionalities. The system controls over 100 droplet microchemostats at once. (b) Scheme of splitting one microchemostat droplet into a waste droplet and a seed droplet of predefined volumes. The procedure is carried out by a careful combination of opening and closing electromagnetic valves triggered by a camera feed of where the droplets are at a given moment. (c) Bacterial growth curve from an exemplary microchemostat with the maximum growth rate (μ_MAX_) shown in the inset. (d) Growth curves from weeks of cultivation of bacteria in droplets with different volume fractions (Δ*V*) of media exchanged periodically at frequency *f*. Gray lines represent theoretical curves depicting Monod’s model. Adapted with permission from ref (4). Copyright 2013 John Wiley and Sons.

Apart from establishing MIC, scMIC, the inoculum effect, or the distribution of resistance in a population of bacteria, there is interest in the process of the emergence of antibiotic resistance. In the most notable microfluidic study,^46^ the authors observed the emergence of resistance in a large microfluidic chamber in which antibiotic concentration formed a gradient. Droplets were also used for long-term bacteria cultivation.^47^ To approach the evolution of resistance with a large-scale experiment in mind, we turned to chemostats: bioreactors in which fresh medium is added continuously, the spent medium is removed, and the droplets are turned into chemostats. The rates of fresh medium addition and old medium removal are identical, yielding a constant volume in the reactor. We designed a system that held over 100, 1 μL-sized droplets simultaneously and circulated them through a closed system of tubing (Figure 7a).^4^ The movement of the droplets around highly oxygenated fluorinated oil even at slow paces allows for the rapid transfer of oxygen from the oil to the droplets,^39^ supporting bacterial growth. At designated time points, the valve system removed aliquots of spent droplets and added fresh medium to the remaining droplets (Figure 7b). Bacterial growth rates were monitored for days (400 h, over 2 weeks, as shown in Figure 7c,d), and we investigated how growth curves changed after adding increasingly concentrated antibiotic. In the future, it may be worthwhile to produce single cell-level microchemostats, a system in which multiple tankers (Figure 3) are circulated, their waste periodically removed, and nutrients replenished. This experimental design would enable studies of the rates of emergence of resistance at unprecedented scale with great multiplexing capabilities. However, current technical problems need to be overcome, such as how to remove and to add medium controllably for each droplet in multiple emulsions.

## 5 Concluding Remarks

Here, we have presented our contribution to microfluidic AST in terms of automating large-scale antibiotic interaction screens and establishing methods for single-cell-resolved population resistance studies, for long-term evolution studies in the presence of antibiotics, and for label-free high-throughput AST in droplets. The microfluidic AST field has recently blossomed, and now, it is possible to assess bacterial resistance to antibiotics quickly enough for the clinical setting. This is not the case for droplets as of yet: to run phenotypic AST in droplets, hours are still needed before the bacteria incubated in the droplets produce a signal strong enough to be detectable by optical or different methods. One can imagine a droplet assay consisting of very small immobilized droplets to monitor the growth of individual cells, but then, the throughput of the method would be limited as constant checking for divisions of individual cells in thousands of droplets seems nontrivial. The only droplet-based assay that used clinical samples for fast (30 min after sample collection) AST read-out was not phenotypic, but pheno-molecular, a hybrid of a genetic and phenotypic assay.^37^ Still, droplet assays are of unparalleled scale. Without droplets, it would not be feasible to screen for rare mutants,^22^ for tens of thousands drug combinations,^23^ or for single-cell resolved resistance.^2,3,41^ When the final goal of a highly multiplexable fast and easy to use droplet AST assay is achieved in the near future, the question that will remain is how best to use such an assay. Will this dreamed assay be usable in the developing world? This Account has highlighted the possibilities and some challenges of droplet-based antibiotic screening. Every advance in this field helps to combat the horrifying prospect of a world with bacteria resistant to every antibiotic that we use against them.
